# Enhancing Wear Resistance and Adhesion of Primer Coatings on Laser-Textured Milled Carbon Fiber-Filled Basalt Composites

**DOI:** 10.3390/polym17091150

**Published:** 2025-04-23

**Authors:** Özer Coşkun, Sinan Fidan, Mehmet İskender Özsoy, Mustafa Özgür Bora, Satılmış Ürgün, Alp Eren Şahin, Taner Yılmaz

**Affiliations:** 1 Naval Air Base Command, Kocaeli 41000, Turkey; ozercoskun_111@hotmail.com; 2 Department of Airframe & Powerplant Maintenance, Faculty of Aeronautics and Astronautics, Kocaeli University, Kocaeli 41285, Turkey; sfidan@kocaeli.edu.tr (S.F.); ozgur.bora@kocaeli.edu.tr (M.Ö.B.); 3 Department of Mechanical Engineering, Faculty of Engineering, Sakarya University, Sakarya 54050, Turkey; 4 Department of Aviation Electrics and Electronics, Faculty of Aeronautics and Astronautics, Kocaeli University, Kocaeli 41285, Turkey; urgun@kocaeli.edu.tr; 5 Department of Mechanical Engineering, Kocaeli University, Kocaeli 41001, Turkey; alperen.sahin@kocaeli.edu.tr (A.E.Ş.); taner.yilmaz@kocaeli.edu.tr (T.Y.)

**Keywords:** laser texturing, wear behavior, milled carbon fiber, basalt composites, coatings, surface treatment

## Abstract

The present study explores the effects of pre-coating on the wear performance of milled carbon fiber-filled basalt composites via laser texturing. Laser texturing was used to change surface topography, enhancing adhesion and wear resistance. Incorporated 0 wt.% and 5 wt.% milled carbon fibers in an epoxy matrix. A fiber laser system was employed for surface treatment, in which power, scanning speed, and pulse frequency were optimized. For pre-coating, an epoxy-based primer was used, and the adhesion and wear performance of the coating was studied using ball-on-disc wear tests. Experimental results demonstrate that laser texturing significantly increases coating adhesion by enhancing the surface roughness and mechanical interlocking. The laser-induced textures displayed mostly square-shaped dimples, reducing practically by around 22% the deformation of the primer coating when used in combination with 5 wt.% carbon fiber milling. The textured surfaces reduced friction noticeably, leading to a decrease of as much as 23% in the coefficient of friction from untreated surfaces. SEM and 3D profilometry analysis indicate that the lower delamination observed in the laser treatment led to optimal coating retention. The original contribution of this work consists of the unique integration of laser surface engineering with pre-coating treatments toward improved tribological performance.

## 1. Introduction

Across a range of industries, including petrochemical, shipping, construction, medical, and defense, coatings are regularly used for wear protection, corrosion inhibition, and sometimes for decorating. Polymer coatings have become increasingly attractive in current times with strong substrate adhesion, high tribological performance, and ease of fabrication. Specifically, high mechanical performance, shrinking during curing, high temperature stability, and high shear strength in epoxy resins have become a high demand in current times. Epoxy resins even best suit complex coating systems with corrosion and chemical inertness [1,2]. With useful properties such as decorating a surface, corrosion protection, sound absorption, insulation, and wear protection, polymer coatings have widespread application in industries such as aeronautics, spacecraft, and medical. Polymer coatings, with performance and durability improvement in coated parts and providing tailor-made options for a variety of applications, are regularly fabricated through a range of preparation processes such as electrostatic powder coating, electrostatic fluidized-bed coating, paint, and flame spraying [3]. With such properties as water/oil repellency, self-cleaning, healing, and corrosion protection, polymer coatings have a key role in altering the interfacial behavior of solid materials. Under a range of environments, coatings with low shear strengths have exceptionally low coefficients of friction and wear rates with high self-lubricity and wear behavior. With such capabilities, polymer coatings stand out in enhancing a range of challenging application surfaces [4,5]. With ease of use, adaptability, and pleasing appearances, coatings such as polymer paints have become routinely used. Polymer coatings have high corrosion, UV, and chemical resistance, in addition to cosmetic improvement. Their performance and protective function go a long way in extending and enhancing the beauty and durability of materials in a variety of industries, including use in consumption, in buildings, and in automotive [6].

Because of their high mechanical properties and high strength-to-weight ratio, polymer composite fiber-reinforced polymer composites (FRPCs) have widespread application in commercial, automotive, and aeronautic sectors [7,8,9]. Traditionally, strengthening has been imparted with aramid, carbon, glass, and Kevlar synthetic fibers, providing high performance assurance [10,11,12,13]. However, with growing concern regarding the environment and legislation, polymer composite natural fiber composites have become in demand with factors such as biodegradability, low density, availability, and efficient use of energy in processing, respectively. In spite of these factors, poor adhesion with a matrix, poor mechanical behavior, and high moisture absorption have been exhibited in natural fibers. Hybridization of synthetic and natural fibers [14,15] is one such mechanism through which one can circumvent these restrictions. Polymer composite demand in the marketplace for a long duration of time has been captured with synthetic fibers such as carbon and glass with high stiffness, high strength, and high durability under mechanical, chemical, and thermal loads, respectively. However, demand for natural fiber-reinforced composites (NFRCs) rose due to the high cost, non-biodegradability, and non-recyclability of synthetic ones. With high mechanical behavior, abundance, competitive price, and reusability and recyclability, natural fibers have been in demand. Because of these factors, NFRCs become a perfect and environment-conscious alternative for synthetic fiber-reinforced polymer composites, in specific cases, considering cost and environment [16]. Positive effects for the environment, naturally derived fibers have increasingly been considered for use in polymer composite strengthening. In these, basalt fibers have become in demand with consideration of their specific behavior and ease in processing. With high modulus, high chemical resistivity, negligible water absorption, and high thermal and sound insulation, these basalt rock-originated fibers have 40–52% SiO_2_ composition. Positioned between S and E glass fibers, they form a cost-effective and high-temperature-resistant attractive reinforcement for high-performance composites [17]. Most of them have widespread use in FRPCs, but most of them have synthetic fibers, which are not biodegradable and cause additional carbon emissions. To mitigate this, a study of natural fiber composites, specifically self-lubricating variants for tribological applications in high wear and tear cases, has been taken up. Pure jute, pure basalt, and jute–basalt bio-composites with nano-graphene fillers (with variable weight fractions) have been fabricated with an epoxy matrix. In tribological tests, a composite with 0.4 wt.% of basalt and graphene at the contact face exhibited a minimum value for specific wear and a value for the coefficient of friction at 1060 rpm [18].

Toorchi et al. [19] examine graphene oxide and nano zirconia as working together to improve the flexural and tribological characteristics of basalt fiber/epoxy composites. A number of composites with different filler loadings were created after the nanofillers’ surfaces were altered to improve their compatibility with the epoxy matrix. The composite with 0.1 wt.% graphene oxide and 1 wt.% nano zirconia had the maximum flexural strength and wear resistance, according to the results. Flexural strength increased by 50% while wear rate and friction coefficient dropped by 67% and 62%, respectively, in comparison to the neat composite. Improved fiber–matrix interfacial bonding was discovered via scanning electron microscopy. For self-lubricating and sliding components that come into contact with metal counterparts, the wear performance of non-toxic, respiratory, non-hazardous natural fiber-reinforced epoxy composites has emerged as a crucial design factor. The epoxy matrix’s poor wear resistance prompted the use of nanoparticles, including graphene nanoplatelets (GNPs), to strengthen it and enhance its tribological characteristics. Vacuum bagging was used to create the BF/EP composites, which demonstrated notable improvements in modulus (74.5%) and tensile strength (42.5%). Ball-on-disk wear testing showed that GNP reinforcement enhanced wear resistance by 60% and decreased friction by 23% [20]. Abu Talib et al. [21] investigated the friction and wear characteristics of glass and basalt fiber-reinforced epoxy composites under various conditions. Two test stages were conducted: the first examined adhesive, abrasive, and erosive wear with sliding against steel, silicon carbide, and sand mixtures, respectively; the second assessed adhesive sliding against a steel counterpart with unidirectional and reciprocating motions at varying pressure-velocity factors and counterpart configurations. Results showed that basalt fiber-reinforced polymer (BFRP) composites exhibited superior wear resistance, particularly under erosive sand conditions and high-pressure-velocity sliding. Boobalan and Sathish [22] investigated the effects of incorporating multi-walled carbon nanotubes (MWCNTs) and silicon dioxide (SiO_2_) in equal proportions, with 0%, 1%, and 2% weight content variations, into a polymer reinforced with natural basalt and synthetic glass fibers. Tribological testing was performed using a pin-on-disc wear test under dry sliding conditions, following ASTM standards. A Box–Benkhen Design (BBD) was applied to design experiments with four variables: filler content, applied load, sliding distance, and velocity. Response Surface Methodology (RSM) and Analysis of Variance (ANOVA) revealed that a 1% filler content resulted in optimal friction and wear properties. Epoxy composites filled with basalt belt, reduced graphene oxide, and paraffin wax (EP/BF/RGO/PW) were successfully developed to enhance both tribological and mechanical properties. Experimental results showed that basalt fibers improved the mechanical strength, while reduced graphene oxide and paraffin wax significantly improved tribological performance. The optimized composite exhibited a 77.5% reduction in coefficient of friction and a 14.4% decrease in wear rate compared to pure epoxy under dry friction conditions. Additionally, the composite’s tensile strength and modulus increased by 75.5% and 100%, respectively, demonstrating the effective synergy of the fillers [23]. Manoharan et al. [24] aimed to develop hybrid friction composites reinforced with recycled basalt and aramid fibers, incorporating various fillers to optimize tribological performance. Dry sliding wear tests were performed using a pin-on-disc rig based on Taguchi’s L27 orthogonal array, evaluating applied load, sliding speed, and fiber content. Grey relational analysis optimized tribological parameters, while ANOVA identified influential factors. The optimal conditions were a 15 N load, 1 m/s speed, and 25% fiber content, which enhanced wear resistance. SEM analysis revealed wear mechanisms, including fiber pullout, matrix cracks, and plateau formation, highlighting the fiber content’s role in improving composite performance. Miniappan et al. [25] investigated the effects of fly ash, basalt powder, and tungsten carbide (WC) on the mechanical and tribological properties of sisal fiber-reinforced epoxy composites. Fillers (5–10 wt.%) were incorporated into a 30 wt.% sisal fiber–epoxy matrix. Basalt powder increased tensile strength by 33.63%, while WC composites (S3) exhibited the lowest wear rates. Basalt powder and WC composites achieved the highest flexural strength (166.4 MPa) and superior hardness. Fly ash with WC and basalt powder enhanced impact strength by 62%. SEM analysis confirmed improved load capacity and adhesion, with ductile failure and wear-resistant features observed.

To achieve clean, active, and abraded surfaces for the best bonding, surface preparation is crucial. Because of their inefficiency, sluggish production rates, and extra cleaning needs, traditional techniques like grit blasting and manual grinding frequently encounter difficulties in industrial applications. By altering surface topography to improve chemical and mechanical adherence, laser surface treatment provides a better option. By removing epoxy resin selectively, this method raises surface roughness and chemical reactivity. Thus, adhesion performance is enhanced by laser surface treatment, which makes it a successful technique for joining composite materials in cutting-edge applications [26]. Laser etching has emerged as a reliable method for bonding composite components, offering enhanced adhesion performance by modifying surface topography. By selectively removing epoxy resin, laser etching increases surface roughness for improved mechanical interlocking and boosts chemical reactivity for stronger chemical adhesion. This technique ensures efficient and durable bonding, making it a preferred choice in advanced composite applications [27]. With increasingly challenging demands, laser-based techniques are rapidly emerging as a promising line of research to deal with the issues and provide a very new path toward optimization of surface properties and performance. In the study by Canel et al. [28], optimization of laser parameters was performed to obtain pits of a specific shape and size. Carbon fiber epoxy composite surfaces were ablated with a 1064 nm Nd:YAG laser. Some important laser process parameters, such as focus position, pulse energy, duration, and number of pulses, were optimized to achieve maximum aspect ratio, circular shape, and minimum thermal defect. It is stated that the optimization process with different parameters can be used to improve some properties, such as pit shapes and geometry, wettability, friction, etc. The research by Jaeschke et al. [29] is on the development of wear resistance mechanisms of tungsten carbide–nickel-based composite coatings. Research has been carried out in such a way that there are so-produced composite coatings through laser cladding that promote wear resistance. The authors developed the wear mechanism and performance of the high-power laser-clad coating and examined the microstructure of the coatings using SEM and XRD. Moreover, the coatings were tested for wear to assess the effectiveness of the coatings. These coatings possess wear-resistant characteristics better than those of the parent material by the hard phases of tungsten carbide, along with a properly bonded nickel matrix. Contuzzi et al. [30] discuss the analysis of how the scanning strategy and key process parameters, like laser power, scanning speed, and frequency, affect the quality of laser-processed composite materials. The composite consists of an epoxy–resin matrix with a woven configuration of carbon fibers reinforced with copper wires, with a particular emphasis placed on the ablation depth and quality of the process area. The processing parameters that yield the ideal results are selected, where deeper ablation is achieved with low speed and high laser power, although the wires get deeply damaged. Yang et al. [31] focused on an investigation of the behavior of carbon–fiber epoxy resin composite under laser irradiation. A 1070 nm laser is used within the current work as a reference to estimate the damage threshold in such composites with laminar structure, and the behavior under laser irradiation is researched. Laser irradiation, on the other hand, was found to affect the surface structure, the thermal properties, and the mechanical properties of the composites described above. Observably, there is a difference in the thermal and mechanical properties as well. It has been found that different degrees of damage and modifications have occurred for these composites under the influence of these laser parameters. Results have shown that the internal thermal stress combined with the gas expansion caused delamination within the composite, giving a quite different effect as compared to bulk composites. Analysis of temperature evolution data, thermogravimetric analysis, and change of composition contributed to understanding the damage mechanism. Gebauer et al. [32] have studied the ablative behavior of CFRPs after surface treatment by means of pulsed lasers. This paper researches the effect of various power, pulse frequency, and scanning speed laser parameters on the processes of ablation, respectively, on the removal rates and surface quality when treated. It was found that a few of the laser parameters optimize the ablative process, which helps remove material effectively from CFRPs without creating thermal damage. Moreover, from the surface quality analysis, it was identified that a few processing parameter sets yield smoother surfaces with less thermal degradation, in addition to fewer defects on the surface. The research also pointed out possible areas for applications of laser surface treatment to improve the adhesion properties of CFRPs for coating or bonding processes. These results have enhanced the interaction of laser energy with ingredients of the composites and shed light on valuable ablative mechanisms. Sharma et al. [33] studied unidirectional carbon fiber reinforced epoxy composites that were processed with a laser beam parallel and perpendicular to the fiber axis using a range of pulse energies. For their morphology, the laser traces were characterized by field FESEM. The ablation process created self-ordered periodic structures on the carbon fiber surface with an average spatial periodicity of 195 ± 45 nm. On the contrary, it resulted in a fine particle morphology, accompanied by numerous voids in the epoxy matrix at all pulse energies studied. Looking at the depth profile of laser tracks made with different pulse energies and reconstructing them in 3D from stereographic images showed that the track depth went up as the pulse energies went up. It is highlighted that the fiber and matrix provide a method for selectively processing components at different ablation rates, depending on the pulse energy used and the scanning speed. Iplikci et al. [34] studied the surface treatment of carbon fiber reinforced polymer (CFRP) composites by using a pulsed infrared nanosecond laser. This study describes the optimization method of removing the top polymer layer and contaminants without causing damage to the carbon fibers. The primary conclusions of the results are that the lower scanning speeds transfer the laser energy as thermal energy, which results in a considerable amount of thermal damage. The high power of the laser damages the fibers. The ideal parameters are said to be at 10 m/s of scanning speed and 30 W of laser power, where there is complete removal of epoxy and a super-hydrophilic surface state. This will be seen to improve the efficiency of adhesive bonding, therefore reducing processing time and costs.

Piscitelli et al. [35] discuss femtosecond laser texturing as a technique toward advancing the adhesion of superhydrophobic coatings (SHC) on composite substrates of CFRP. The laser-textured surfaces, in comparison with the untreated surfaces, demonstrate more sustained superhydrophobic behavior, even after successive cleaning processes. The results emphasize laser texturing as a green, efficient, yet trustworthy surface treatment process, capable of enhancing the bonding performance of SHC on composite substrates while ensuring sustained functional stability. A KrF Excimer laser (248 nm) was used for the purpose of enhancing the adhesion of paints on treated surfaces. Laser-treated substrates, in comparison with as-received as well as sand-papered samples, demonstrated higher adhesion. Such improvement in adhesion has been explained by the effective removal of surface impurities, decrease in polymer chain scission, the higher occurrence of surface-active groups, and higher surface roughness, all of which lead to better coating performance, as well as higher durability [36]. Mahdy et al. [37] demonstrate that incoherent UV light treatment at 254 nm significantly enhances the adhesive bonding strength of autoclave-cured T700 CFRP composites by 75% compared to untreated samples and approximately 10% over NIR laser-textured surfaces. Using a 46 W germicidal UV lamp and a 1064 nm IR nanosecond pulsed laser, surface treatments were evaluated through wettability and bonding tests. Results show UV treatment is a cost-effective method to activate CFRP surfaces, improving adhesion by reducing water contact angles. Alsarani et al. [38] evaluated the effect of femtosecond laser-generated surface patterns on the enclosed mold bond strength (EM-SBS) of resin composites to zirconia (ZrO_2_), compared with tribochemical silica coating (TBC). Among the five groups, the cross-patterned laser-treated group (G4) showed the highest EM-SBS, even after 5000 thermocycles. Laser-treated surfaces exhibited greater roughness and lower contact angles. Two-way ANOVA confirmed significant effects of surface treatment and aging. Femtosecond laser texturing presents a promising alternative to conventional zirconia surface conditioning techniques.

Currently, sandblasting, chemical treatment, or plasma etching are used primarily to enhance adhesion. Though these methods are typically more time-consuming, more expensive, or involve extra cleaning procedures, laser texturing provides a more efficient, more accurate solution by selectively modifying the surface topology in a way that promotes the mechanical interlocking along with chemical bonding with coatings. The potential of this method in producing highly controlled surface patterns in composite materials may prove to be vital in surface-functionality-driven applications in aerospace, as well as in the automotive sector. In industrial contexts, in comparison with the literature, the combination of laser surface modification with basalt fiber composites filled with milled carbon could represent a new way of generating high-performance lightweight composites with superior strength and environmental durability. Such composites can find use in structural components under surface impact, where traditional materials are not able to meet the requirements for adhesion or long-term performance. In aircraft components, automotive body coatings, or advanced materials in the construction industry, this technique can prove useful by being a green, low-cost alternative in comparison with traditional synthetic fiber-reinforced composites.

The present study explores the effect of laser texturing on the adhesion and wear resistance of primer coatings applied to milled carbon-filled basalt fiber composites under varying laser parameters, including power, speed, and frequency. After laser texturing, primer coatings were deposited, and their adherence to the composite surfaces was assessed using pin-on-disc wear tests. Post-test evaluations were conducted through 3D profilometry, optical microscopy, and scanning electron microscopy (SEM) to examine the surface characteristics and wear mechanisms. However, to the best of the authors’ knowledge, while numerous studies have explored laser surface modification of CFRPs, the combined effects of laser texturing and functional filler additives on basalt fiber-reinforced epoxy composites containing milled carbon fiber fillers remain underexplored. In particular, insufficient research has concentrated on how the surface topography introduced by a laser, in combination with the existence of carbon fibers milled within the matrix, affects the adhesion properties and wear behavior of primer coatings in such hybrid composites. In this context, basalt fiber-reinforced epoxy composites modified with milled carbon fiber fillers represent a promising class of materials due to their favorable mechanical performance, thermal stability, and improved conductivity. The incorporation of milled carbon fibers enhances the composite’s interfacial bonding potential while also contributing to the dissipation of heat generated during laser processing, potentially leading to more uniform and controllable surface textures. The synergistic influence of laser texturing parameters (e.g., power, scanning speed, frequency) and milled carbon fiber addition on the resulting coating adhesion and wear resistance has not been systematically investigated. Such findings are anticipated to provide novel insights into developing wear-resistant composite materials suitable for applications in aerospace and automotive industries, where improved environmental resistance and durability are critical. This approach integrates innovative laser-based surface modification techniques with functional coating applications, contributing to the advancement of composite materials for high-performance engineering.

## 2. Materials and Methods

### 2.1. Materials and Manufacturing of the Composites

In the present work, a hybrid composite of basalt fiber reinforcement and milled carbon fiber (MCF) fillers at a content of 5 wt.% was developed using epoxy as the matrix material. Basalt fibers have high tensile strength, high-temperature stability, and chemical resistance, so they are ideal for use in structural parts due to the mechanical and environmental stresses they are exposed to. The reinforcement used was a 200 g/m^2^ bidirectional basalt fabric (Zhejiang GBF Basalt Fiber Co., Ltd., Dongyang City, China), featuring a density of 2.60–2.63 g/cm^3^, fiber diameter of 13 µm, tensile strength of 3100 MPa, elastic modulus of 88–92 GPa, and an elongation at break of 3.5%. Balanced warp and weft yarn densities (10 threads/cm) ensured isotropic mechanical behavior in the plane of the fabric. The milled carbon fiber fillers (ELG Carbon Fibre Ltd., Bilston, UK) had a nominal fiber diameter of 7 µm, a length of 80 µm, a particle size of 80 µm, a tensile strength of 3470 MPa, and a modulus of 246 GPa. Their addition was to increase interfacial adhesion and wear resistance by strengthening the matrix and facilitating improved load transmission. Before fabricating, the MCFs were dried at a temperature of 80 °C for 24 h to evaporate the water content. Preparing the epoxy matrix involved the mechanical dispersion of the MCFs in the resin to create a uniform mixture. Then, the hardener was added and stirred thoroughly. This mixture was then applied layer by layer onto basalt fiber fabric using the brushing and roller method in order to build up the desired thickness in the laminate. The lay-up was then cured in the oven under controlled temperature and time conditions. The combination of high-modulus basalt fibers and well-dispersed carbon fillers within the epoxy matrix was designed to improve the tribological performance of the composite, especially under laser texturing and pre-coating conditions. This hybrid reinforcement approach promotes enhanced interfacial bonding, mechanical durability, and improved resistance to surface wear and coating delamination, providing a strong foundation for investigating the effects of laser surface modification and primer adhesion.

### 2.2. Laser Processing

**Figure 1:**
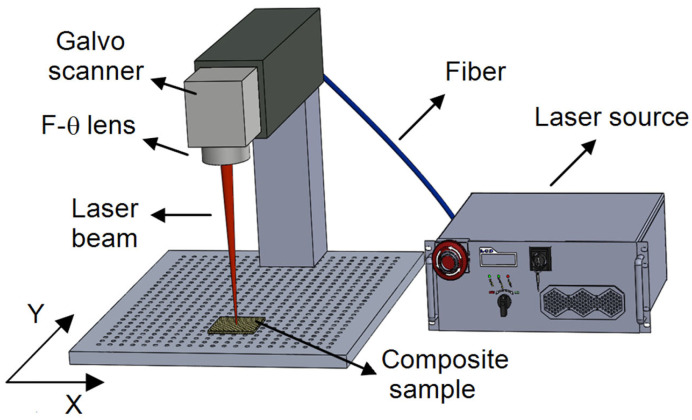
Schematic of the fiber laser system used in the experimental design.

This research extends our previous studies by systematically exploring the basalt fiber-reinforced composite properties, which contain exceptional thermal and mechanical properties. Figure 1 shows a laser system used in texturization. The laser system employed in research is a fiber laser in pulse mode, with a wavelength of 1064 nm. The average power of this laser, with a maximum power of 100 W, can be adjusted by changing the duty cycle of the PWM signal between 10% and 100%. Furthermore, the frequency of the fiber laser can be varied between 20 kHz and 200 kHz, while the maximum processing speed can reach 9000 mm/s. The laser machining process was carried out in an atmospheric air environment.

**Figure 2:**
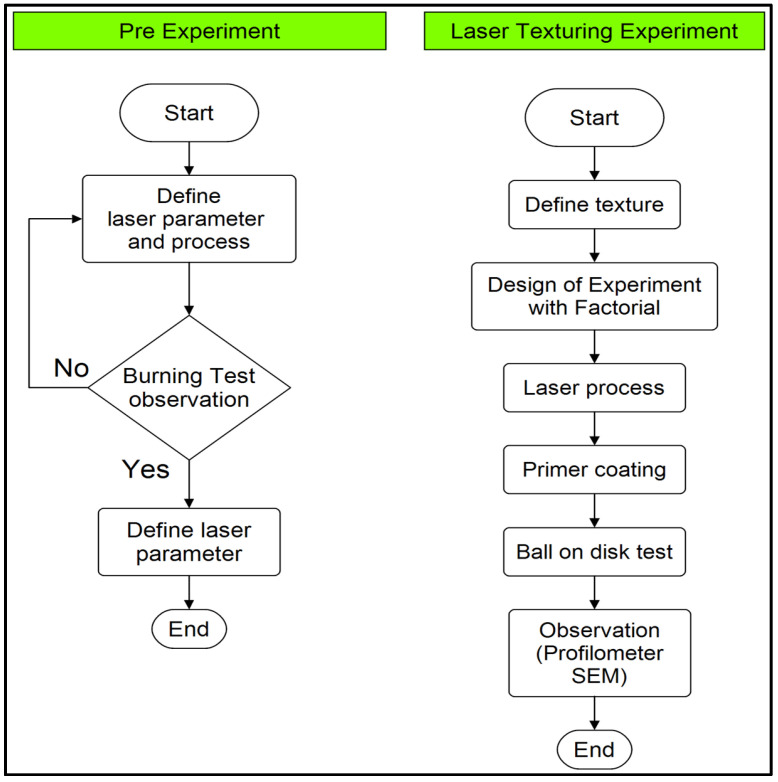
Flow diagram of the laser processing sequence of milled carbon fiber-filled basalt composites.

In this respect, the common characteristics of the laser system, in terms of power level, scanning speed, and pulse frequency, were considered, and their systematic change was made to investigate their influence on the composite material. The galvo head in the system allows precise beam steering by moving in the X and Y axes, enabling high-speed and accurate texturization of the surface. The main goal was to determine the most effective laser settings that provide the appropriate depth of texturization while achieving the highest pre-coating adhesive strength. Figure 2 presents the flow diagram illustrating the laser processing of milled carbon fiber-filled basalt composites.

The methodology of the study contained various critical steps that ensured an in-depth comprehension of the laser texturization process on milled carbon fiber-filled basalt composite samples. The textured prepared composite samples were then laser-textured using a fiber laser system with systematic variations of the process parameters: laser power, scanning speed, and pulse frequency. The selected optimal parameters were used, and the composite surfaces were characterized using a non-contact laser profilometer, optical, and scanning electron microscopy for texture quality determination, depth, and uniformity of the laser-processed surfaces. This was followed by primed composite surfaces that were further submitted to a primer coating process, and whose adhesion to the textured surfaces was assessed to ensure that texturization enhanced the bond strength of the primer coating. Such an approach provided the best set of parameters for laser texturization with high-quality surface modification and minimum thermal damage of the composite material.

This includes a prime focus on the ability of visual analysis of the smoke so as to lead to the correct texturing results required over advanced composite materials through the laser processing parameters. The smoke generated during carbon fiber conversion also provides useful data for greyscale analysis in order to optimize laser settings. It was concluded that the minimum smoke emission was observed at a laser power of 10 W, scanning speed of 2000 mm/s, and frequency of 20 kHz. With respect to the depth of the produced lines, such parameters were considered to be the most feasible processing parameters for texturing. The flowchart shows the testing process prior to laser operation. The process starts with the determination of the laser parameters, followed by readjustment of the parameters based on the observations of the burn-in test. If a suitable result is not obtained during the combustion test, the process is repeated by re-adjusting the parameters. In this context, as a result of the experiments, it was determined that the minimum smoke emission was observed at 10 W laser power, 2000 mm/s scanning speed, and 20 kHz frequency. At these parameters, the pulse duration was calculated as 5 microseconds (µs). Considering the depth of the produced lines, these parameters are considered to be the most suitable laser settings for surface texturing.

**Figure 3:**
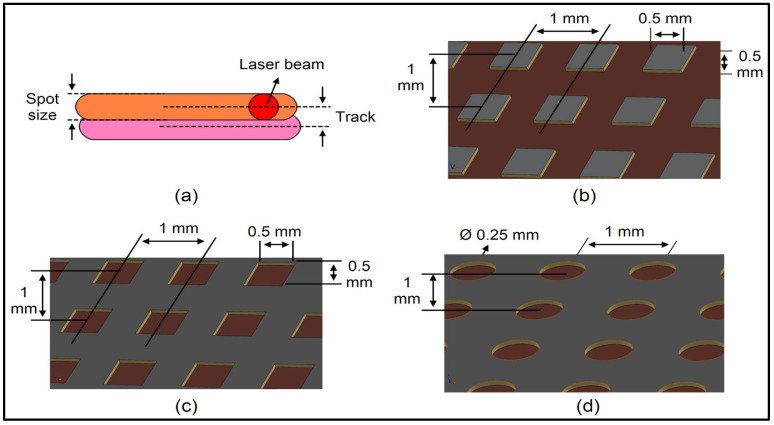
Schematic of (**a**) laser parameter used for texture surface scans, (**b**) T1 texture dimensions (square), (**c**) T2 texture dimensions (square dimple), and (**d**) T3 texture dimensions (circle dimple).

Increasing the laser power above 10 W caused burns on the polymer surface, and significant carbonisation and high smoke emission were observed during the process. This caused undesirable damage due to the excessive heat exposure of the surface. The burning effect negatively altered the surface morphology and disturbed the homogeneity of the texturing process. On the other hand, when the laser power was reduced below 10 W or the scanning speed was increased (energy density decreased), no obvious texturing of the polymer surface was achieved. This low energy density prevented sufficient material removal on the surface, resulting in no visible change on the surface. The wear testing mentions three different sizes of textures that provide several benefits, namely T1, T2, and T3, as illustrated in Figure 3. The surface patterns shown in the figure will be created by a grating scanning method using a fibre laser. As shown in (a), the desired geometry will be obtained on the surface by moving the laser beam to have a certain spot size. Among the patterns produced by this method, (b) T1 represents a structure in which square-shaped grooves are formed at regular intervals on the surface. (c) T2 has a surface texture in which the edges of the square grooves are modified to have smoother transitions. (d) T3 has a different surface morphology designed with round grooves. During these processes, laser parameters (power, speed, scanning distance, etc.) can be optimised to control the mechanical and tribological properties of the surface. With the application of different geometric patterns, performance criteria such as friction, wear resistance, and adhesion properties of the surface can be improved, and surface designs suitable for the application can be developed. The presence of these texture structures will increase the wear resistance of surfaces manufactured from basalt composites, as well as improve the material’s performance due to a more homogeneous distribution of stresses across its surface. As a result, surfaces become more durable and more resistant to wear. This is particularly relevant in wear tests like ball-on-disc types.

As a result of preliminary experiments, the best laser parameters were determined, and textures were created using these parameters. The laser parameters used to create the T1, T2, and T3 textures shown in Figure 3 were 10 W power, 2000 mm/s speed, and 20 kHz frequency. These parameters are the ones that create a trace on the surface where the grazing effect is minimized. In order not to damage the composite surface, these parameters will be used as repeated pulses. For four different amounts of basalt composites with chopped carbon and three different textures, an experimental design with the parameters shown in Table 1 was used.

### 2.3. Pre-Coating Application

The pre-coating stage was exploited to enhance adhesion and laser-textured basalt composite wear resistance. The samples underwent a cleaning stage with isopropyl alcohol (IPA) in an attempt to remove any residues contaminating the surface. Compressed drying with air ensured proper drying. It was a key stage in an attempt to remove any contaminating residues and impurities at the surface that could hinder the adhesion of the primer. A two-component epoxy primer (MIL-PRF-23377, MABAYCO, İstanbul, Turkey, equivalent grade of an aircraft primer) was sprayed with an HVLP (High Volume Low Pressure) spray system. All operations are conducted under environmentally conditioned temperature and humidity (21–25 °C, <65% relative humidity) in an effort to exclude moisture trapped in paint during drying. The primer was sprayed in a uniform, even, target wet-thickness of 15–25 µm, measured with a dry-film-thickness (DFT) gauge according to ASTM D7091 [39], requirements. After application, samples with 10–15 min flashing off for evaporation of solvents underwent 24 h at room temperature, and a post-curing at 60 °C for 2 h for maximization of adhesion and full crosslinking. Twenty-four hours at room temperature and 60 °C for 2 h post-curing for maximization of adhesion and full crosslinking for its selection in balancing durability and mechanical integrity of coatings, and not developing thermal stress in the composite matrix, was adopted. The pre-coating treatment was homogeneously imparted onto all laser-textured samples, and homogeneity in adhesion behavior for a range of surface topographies and milled carbon contents was assured. Effects of pre-coating regarding wear and adhesion behavior were subsequently examined in wear track tests and adhesion tests.

### 2.4. Wear Test

**Figure 4:**
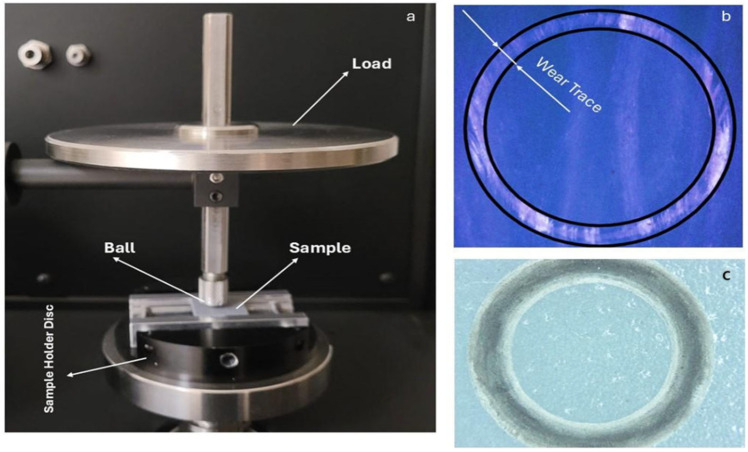
(**a**) Photograph of test device, (**b**) schematic photo of wear trace, and (**c**) wear track microscope image of real worn sample.

The sliding wear tests were performed using the Nanovea T50 tribometer (Irvine, CA, USA) according to ASTM G99 test standard (ball on disc test type). The test samples were subjected to a normal load of 20 N and a rotation speed of 1 m/s at a rotation radius of 4 mm for a distance of 150 m at ambient temperature and humidity. The photograph of the wear test machine and the schematic photo of the wear trace are given in Figure 4a,b. An example microscope image of the actual wear trace is given in Figure 4c.

### 2.5. Profilometer Surface Analysis

The Nanovea PS-50 non-contact laser profilometer was used to perform surface analysis of scratch traces on the laser-textured milled carbon fiber-filled basalt/epoxy composites. This is a specialized profilometer for the accurate characterization of surface roughness and morphology of metallic, ceramic, polymeric, and composite materials. This study carried out a detailed analysis of the depth and width of the wear tracks, as well as the three-dimensional morphology of the wear tracks of ball-on-disc scratch tests, departing from traditional roughness parameters like Ra (average roughness) and Rz (maximum roughness depth). The scan of the scratch crater (approximately 12 mm × 12 mm) was made using the profilometer, making sure the scratch was contained within the scan area. Surface scanning was conducted at a spatial resolution of 20 µm for the X and Y directions, and the acquisition frequency was set to 1000 Hz. Mountains Technology DigitalSurf Software (version 6.2.7487) was used to process the images further and to accurately characterize the wear tracks. This accuracy was important in assessing the effect of laser-texturing parameters and carbon fiber content on surface adhesion and wear resistance behavior. The portion of the deformed area due to the primer coating in relation to the total area was measured with the help of an optical profilometer. The tool provided surface topography measurements of high resolution in a non-contact mode, thus permitting accurate identification as well as measurement of the deformed area on the coated surface. The three-dimensional surface profile data were used to identify the deformed regions and intact surface, and their proportion in relation to the total area analyzed was measured accurately.

### 2.6. Scanning Electron Microscope (SEM) Analysis

The wear mechanisms of the tested samples and the morphologies of the worn surfaces were analyzed in detail using SEM photographs taken using a JEOL brand SEM device (Tokyo, Japan).

## 3. Results

### 3.1. Surface Morphology and Wear Track Analyses

**Figure 5:**
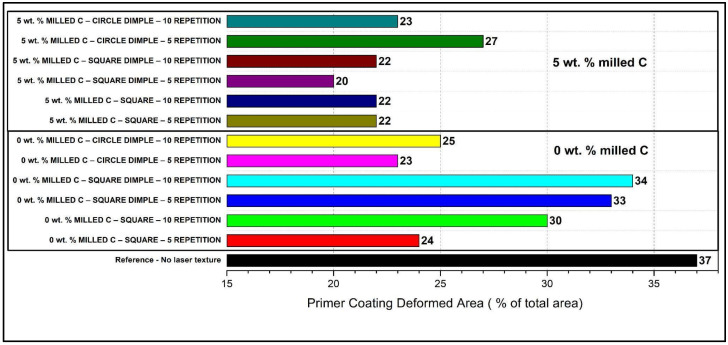
Comparison of % primer coating deformed area versus total area.

Figure 5 illustrates a comparative analysis of the contributions of laser-textured surface structures, milled carbon fiber compositions, and laser repetitions towards primer coating deformation in basalt–carbon fiber composites. The investigation offers prospects for understanding and refining adhesion and mechanical attributes as a function of surface modification through distinct laser textures and milled carbon fiber fillers. The impact of milled fiber content is clearly demonstrated in the primer coating deformation in all samples that were tested. In contrast, the sample without laser treatment (reference) has the highest value for deformation percentage, which represents poor adhesion behavior. While all laser-textured surfaces outperform the reference, the 0 wt.%, some 1 wt.% milled composite samples retain the same high deformation percentages, indicating that the use of milled carbon fibers alone cannot provide the necessary reinforcement effect of laser surface treatment. The lack of carbon fibers leads to insufficient matrix adhesion, even with improved roughness due to laser scribing and possible mechanical anchoring sites. For example, the stress distribution and mechanical interlocking offered by square dimple structures may contribute to the consistently superior performance compared to circular dimples. Circular dimples significantly increase adhesion in comparison to the reference. However, their deformation percentages are still comparatively higher than square dimpled surfaces. Trailing more laser repetitions from 5 to 10 has a minimal effect, through increased roughness, on the adhesion, and this impact is especially pronounced in samples free of milled carbon fibers. On the other hand, for samples with 5 wt.% milled carbon fibers, subsequently increasing laser repetitions yields only slight marginal returns. It must be noted again that above a certain critical roughness (of ~nm), increasing the roughness will not effectively contribute to improved adhesion (it may eventually backfire due to (macro-)ablation). Samples containing 5 wt.% milled carbon fibers demonstrate consistently low deformation percentages, underscoring the significant reinforcing nature of carbon fibers with respect to interfacial adhesion and mechanical integrity. This improvement in these samples is linked to better load transfer and improved fiber–matrix adhesion and structural stiffness. The 5 wt.% square dimple texture milled carbon fiber is still the best result, which highlights the importance of geometric shaping and intra-fiber addition for the actual mechanical adhesion. Square dimple structures generally minimize deformation due to larger mechanical anchoring and maintenance of the primer integrity, while circular dimples, although helpful, do not provide effective mechanical anchorage. Square textures without dimples (albeit better than the reference case) still underperform compared to the dimpled textures, emphasizing the need for localized anchorage points. The basalt composite surfaces textured by a fiber laser are predicted to exhibit a number of advantages in wear tests. Micro and nanoscale structures realized on the surface enhance the surface area, resulting in better mechanical interlocking, especially useful in adhesion. The microstructures also enhance resistance against friction forces. Surface texturing also enhances surface energy, thus increasing wettability as well as resulting in better bonding in coating and adhesive processes. The decreased coefficient of friction from these textures results in reduced friction between moving components, which is helpful in wear performance [40,41,42]. Parallel track patterns lead to more even stress distribution, thus leading to more consistent resistance against friction. Square cell structures improve mechanical bonding by increasing surface area and load-bearing properties. They are also capable of prolonging surface lifetime by preventing stress concentrations as well as more even loading. In circular cell patterns, lower forces of friction lead to reduced wear, while their geometry can facilitate a lubricating effect, further reducing friction [43,44,45]. As explained in Ref. [46], laser surface texturing (LST) has a strong impact on tribology in relation to the curve of frictional behavior, but more research is required under conditions of dry friction. Wang et al. [46] explored the tribological performance of PTFE/Kevlar fabric composites on LST steel surfaces with round dimples (diameter of 100 µm, area density of 30%, depth-diameter ratio of 0.1). They experienced a 64% decrease in friction as well as an 82% reduction in wear at optimal LST values. These were realized through the development of a transfer film as well as due to the capability of the micro-dimples in trapping debris as well as lubricant. Ref. [44] corroborates artificial surface textures in decreasing friction and wear resistance, acting as reservoirs of lubricant subject to their design. Such factors as variations in texture area, aspect ratio, as well as in shape, however, make it challenging in standardization as well as comparison of texture performance from study to study. Atlioglu and Kaynak [47] experimented with untreated as well as sandblasted PPS/CF laminates and the laser and plasma treatments, testing adhesion performance through cross-cut tests. All the treated surfaces maintained a GT0 adhesion value. The better adhesion was attributed to better mechanical interlocking from the surface roughness, as well as due to higher surface energy by means of better wettability.

**Figure 6:**
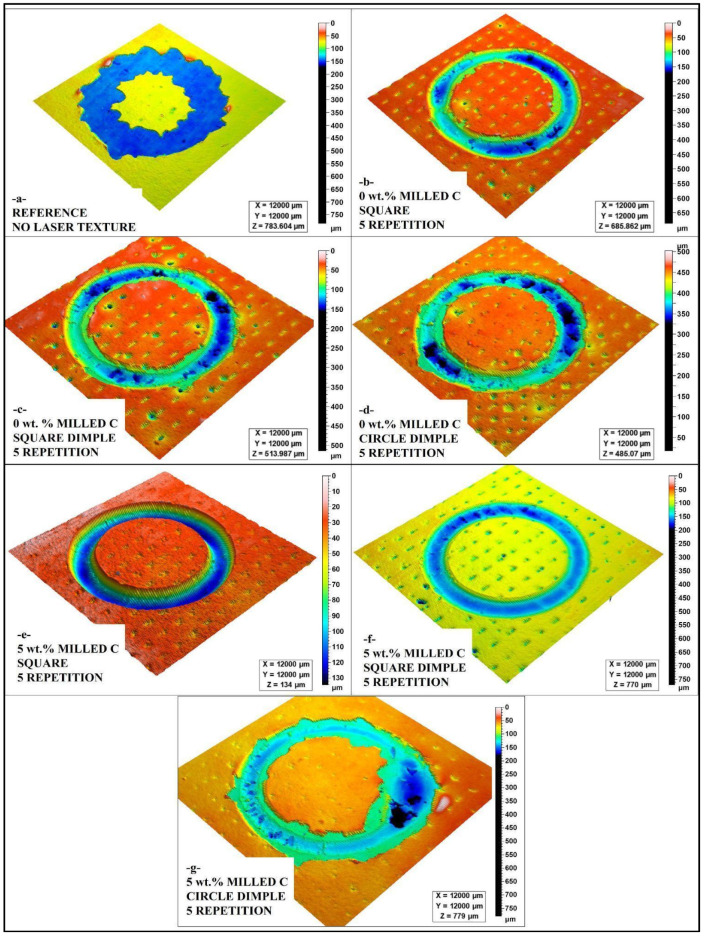
Comparison of 3D surface morphology 5 repetition laser treated surfaces (**a**) reference, (**b**) 0 wt.% milled C square, (**c**) 0 wt.% milled C square dimple, (**d**) 0 wt.% milled C circle dimple, (**e**) 5 wt.% milled C square (**f**) 5 wt.% milled C square dimple (**g**) 5 wt.% milled C circle dimple.

In Figure 6a, the laser free-reference textured surface reveals poor adhesion strength with no feature of mechanical interlocking. Poor adhesion between substrate and coating, and, in consequence, poor adhesion with reduced adhesion state is developed with relatively even morphology. There is no roughness in the surface and, consequently, no strong contact at interfaces, and, in consequence, a high level of deformed primer coating in adhesion tests.

In Figure 6b, a square textured surface at 0 wt.% milled carbon and five laser repetitions exhibits a satisfactory adhesion improvement. The textured surface forms a larger adhesion contact area, but reduced milled carbon fillers constrain interfacial stiffness. The adhesion performance is not satisfactory; yet, in comparison with samples with a carbon filler, the polymer matrix cannot produce enough bonding stability alone.

In Figure 6c, square dimple roughness over 0 wt.% milled carbon even enhances adhesion strength over a simple square roughness. Dimples allow for increased mechanical interlocking, offering increased anchorage locations for the primer coating. Yet, with no milled carbon, the adhesion of the coating is even less strong in comparison with variants with added carbon. Deformation in adhesion testing reveals that roughness is a positive factor, but that a reinforcing phase is critical for the best adhesion.

In Figure 6d, it can be noticed that the dimple texture in 0 wt.% milled carbon is not uniform in adhesion performance. With localized adhesion regions developed through dimples, poor strengthening creates less integrity at the interface at a structural level. The spherical dimple shape cannot generate a similar level of interlocking between substrate and dimple, and adhesion is variable in terms of its intensity, creating high deformability in coatings.

In Figure 6e, the square is textured with 5 wt.% milled carbon reveals the greatest adhesion strength for all of the examined configurations. Higher contents of carbon have a strong impact on enhancing interfacial adhesion and minimizing primer coating deformation. Maximum mechanical interlocking is guaranteed through a textured surface, and therefore, such a variant is most effective in enhancing adhesion and wear resistance. 

In Figure 6f, a square dimple texture is added to 5 wt.% milled carbon for even improved adhesion performance. High carbon and uniformly spaced dimple structures make an ideal substrate for the adhesion of a primer. In adhesion tests, minimum deformity in coatings yields a strong and durable adhesion at the interface.

In Figure 6g, circle dimple texture functions effectively but with a marginally reduced adhesion strength compared to square dimple textures. Square dimple textures exhibit a square-shaped geometry in a circular arrangement, producing less mechanical locking, and hence, a marginally increased deformation in adhesion tests is noticed. Nevertheless, even with a marginally increased one, an improvement over non-filled samples is noticed with increased contents of carbon.

Overall, the analysis of Figure 6 shows that milled carbon fillers and laser texturing have a significant role in improving the adhesion strength of pre-coating. Square dimple texture is uniformly observed to exhibit high mechanical interlocking, most noticeably in 5 wt.% milled carbon samples. Observations confirm that a correct mixture of laser texturing and a correct filling phase is important in creating high-performance coatings with high durability and wear resistance.

**Figure 7:**
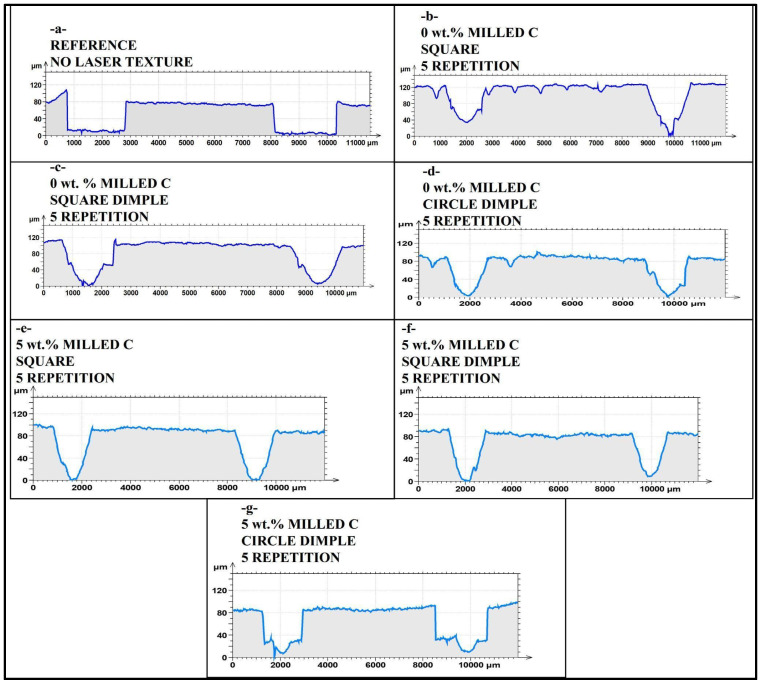
Comparison of cross-section of wear track from mid-point of 5 repetition laser treated surfaces (**a**) reference, (**b**) 0 wt.% milled C square, (**c**) 0 wt.% milled C square dimple, (**d**) 0 wt.% milled C circle dimple, (**e**) 5 wt.% milled C square (**f**) 5 wt.% milled C square dimple, and (**g**) 5 wt.% milled C circle dimple.

In Figure 7a, a relatively shallow and wide wear track for an untextured laser reference sample signifies poor adhesion strength and poor wear life at the stage of pre-coating. With no mechanical interlocking feature in its surface topography, minimum adhesion between substrate and coating occurs, and delamination susceptibility is, therefore, increased. With a relatively constant track depth, uniform loss of material is displayed, in compliance with poor adhesion.

In Figure 7b, the 0 wt.% milled carbon (M0) square laser textured wear track is deeper and relatively rougher when compared with the reference. Incidental laser roughness generates additional surface area, and hence relatively increased adhesion strength, but no milled carbon strengthening holds back durability of the coating, and significant wear depth is encountered. Edge roughness in terms of localized loss of material in terms of ridges and troughs indicates partial delamination of coatings.

In Figure 7c, the square dimple microstructure in M0 shows a well-established wear track profile with increased variation in depth. Dimples cause mechanical interlocking and, hence, contribute towards holding the coating partially in position. However, in lack of any strengthening, adhesion is not perfect. The presence of sharp fluctuations in depth signifies that peeling off of the coating is localized, and processes of wear depend on concentrations of stress at dimple borders.

In Figure 7d, the dimple circle texture in M0 shows a less extreme variation in depth when compared with a square dimple. There is a variation in contact stress in a geometric manner, and a less uniform track is generated, but it is a little less uniform when compared with it. Poor adhesion, however, is present, and it can be seen through relatively deep wear penetration. Loss in adhesion must be in localized regions with high concentrations of stress through a lack of mechanical interlocking.

In Figure 7e, the square textured with 5 wt.% milled carbon (M5) reveals a shallow and uniform wear track between all samples under testing. High levels of carbon and laser-formed roughness together contribute to a high adhesion strength and a minimum peeling off of coatings. Depth uniformity in profile indicates that the coating is effectively resisting wear propagation, and it confirms the best interfacial adhesion developed with such a configuration.

In Figure 7f, square dimple texture imparts additional adhesion improvement and wear in M5. Wear track is shallow with little loss of material, suggesting high performance in terms of bonding. High mechanical interlocking is imparted in dimples, and delamination is effectively checked, and coatings are kept intact. This configuration is a perfect combination of strengthening and patterning of a surface.

In Figure 7g, the circle dimple texture in M5, even with its effectiveness, suffers a little deeper wear track compared to the square dimple texture. There is no such mechanical interlock in a rounded shape, and for that reason, a little more material is taken out, but with a high milled carbon content, adhesion and wear life become much improved in relation to M0 types.

Overall, the comparative analysis in Figure 7 reveals strong laser texturing and milled carbon fillers influence over the wear track shape and adhesion strength of pre-coating. Square dimple textures consistently produce the best performance, particularly in 5 wt.% milled carbon samples, in terms of optimized wear and durability of coatings. All observations validate that both laser texturing and the contents of fillers have to be optimized in a manner that yields the best adhesion and tribological performance in polymer composite materials. This comparative study reveals that laser texturing’s success in generating more consistent and shallow wear tracks is highly reliant upon the texture geometry and composite reinforcement.

Additional analysis of the worn regions from profilometer scans accounted for the mechanical material removal as well as the delamination of the coating and exposure of the substrate. Quantitative analysis of the measurements confirmed that the untreated reference sample, with no laser texturing, had the greatest total worn area (118,765 µm^2^) and hence the maximum coating asperity failure resulting from poor primer adhesion under tribological loads. All of the laser-textured surfaces, conversely, had a notable decrease in the total worn area. In the 0 wt.% milled carbon category, the square pattern had the least wear (65,222 µm^2^), followed by the circular dimple pattern (70,203 µm^2^) and the square dimple design (87,827 µm^2^). However, compared to the untreated surface, the wear remained notably lower. In the case of the 5 wt.% milled carbon category, the pattern followed the same trend, with square pattern textures having the least worn area (73,348 µm^2^), followed closely by the square dimples (75,245 µm^2^) and square patterns (84,963 µm^2^). The observation indicates that laser surface texturing leads to increased mechanical interlocking, increased primer hold, and increased uniformity in the distribution of tribological loads, hence improving the wear resistance overall. The diminution in wear areas in the case of all of the laser-treated specimens, irrespective of the carbon reinforcement, lends credence to the fact that topography engineering of the surface is an important aspect of increasing the durability of the coating under sliding conditions.

### 3.2. Coefficient of Friction Results

**Figure 8:**
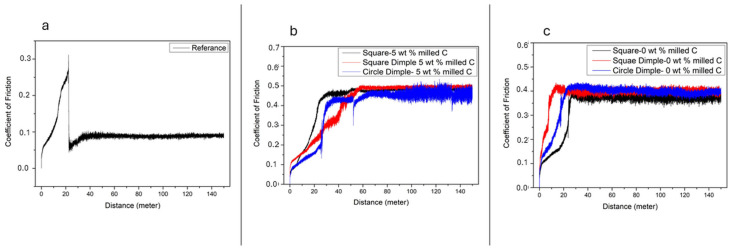
Coefficient of friction value after 5 repetition sliding test; (**a**) Reference sample, (**b**) 5 wt.% milled C, (**c**) 0 wt.% milled C.

**Figure 9:**
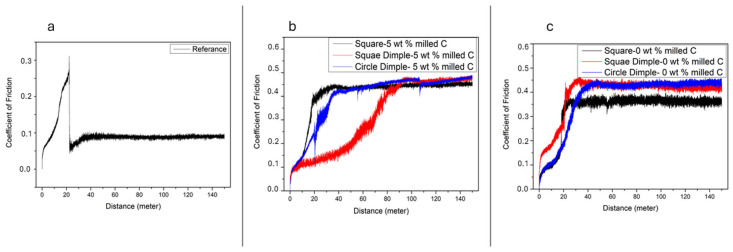
Coefficient of friction value after 10 repetition sliding test; (**a**) Reference sample, (**b**) 0 wt.% milled C, (**c**) 5 wt.% milled C.

Figure 8 and Figure 9 show the changes in the coefficient of friction on the sample surfaces during the test. The current graphs clearly show that the coating adhesion with the reference sample has failed. The instantaneous coefficient of friction changes graphs given in Figure 7a and Figure 8a clearly show this. It is seen that the friction coefficient value suddenly dropped at the 20th meter of the experiment as a result of the coating breaking away from the surface. In Figure 7b,c, when 5 repeat laser shots are applied to the sample surfaces, it is seen that the coefficient of friction becomes irregular in both 0 wt.% C samples and 5 wt.% C samples for circle dimple laser pattern. This is clearly seen, especially in the coefficient of friction curve of 5 wt.% C samples. However, it can be easily stated that the coating has a positive effect on the adhesion of the sample surface for other surface patterns. The effect of 10 repeated shots on the change of the coefficient of friction is seen in Figure 8b,c. While irregularities are still noticeable in the friction coefficient curve for the 0 wt.% C sample, it is seen that this situation improves in the 5 wt.% C composite sample. On the surfaces where the square laser pattern was applied, the coefficient of friction curve, which was smooth on the surfaces where 5 repeat laser shots were applied for 5 wt.% C samples, were disrupted on the surfaces where 10 repeat laser shots were applied.

### 3.3. SEM Examination of Wear Tracks

**Figure 10:**
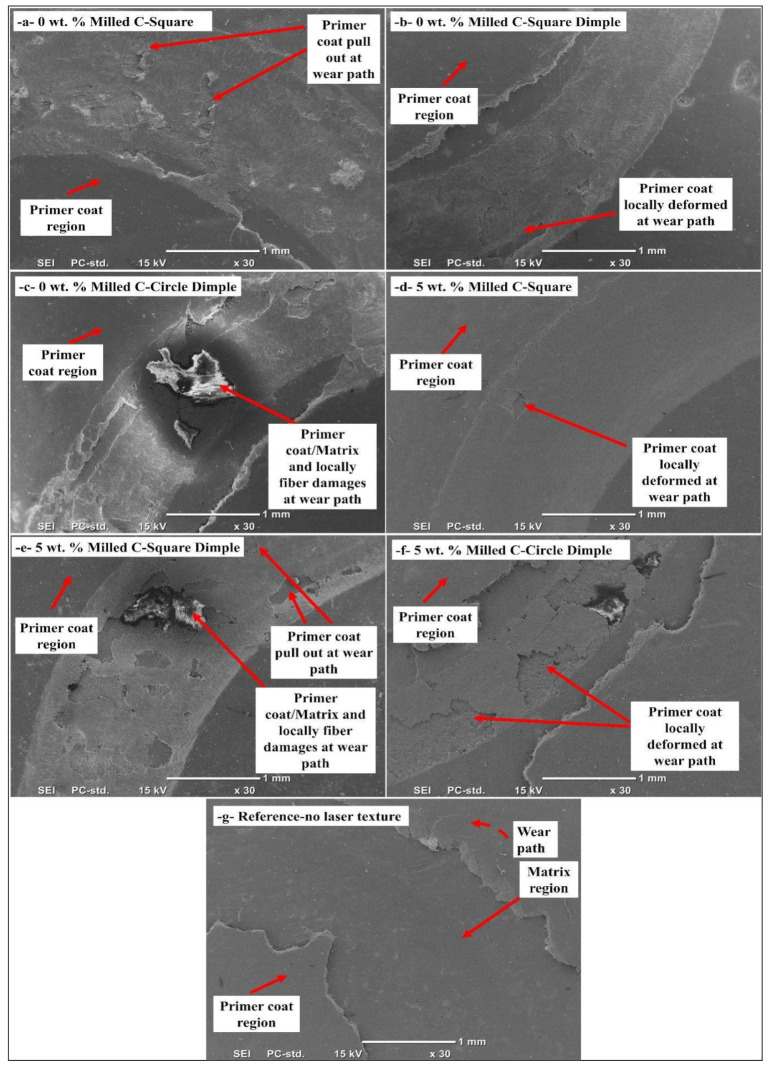
Wear surface of primer-coated and various laser-textured composite samples with 0% and 5% milled carbon fiber-filled (30×).

The morphology of the wear track of laser-textured and primer-coated composite specimens was measured using pin-on-disk wear tests and is presented in Figure 10. The images also convey the perspective about tribological performance of different laser surface textures (square, square dimple, and circular dimple) on composite surfaces with 0 wt.% and 5 wt.% milled carbon fibers. The characteristics of the worn tracks from lapping the laser-textured surface against the primer coating and composite matrix reveal the mechanisms taking place in sliding wear. The wear track could be divided into two main areas. The undeformed region of the primer coat is the region where the indenter has not significantly interacted with the surface, and has allowed the coating to retain its integrity without appreciable deformation. Unlike that of the deformed primer coat area, which forms due to the applied contact stress during the wear testing phase, leading to degradation and detachment of top coats from the composite underlying structure. In this region, the deformation distribution and size highly depend on the laser texturing pattern, the presence of milled carbon fibers, and the adhesive strength of the primer coating. For the square laser-textured models, primer pull-out is largely noted at the wear route. Localized deformation in the coating and composite matrix for the 0 wt.% milled carbon fiber sample. This indicates that the square laser texture fails to mechanically anchor the coating and thus does not provide enough of an anchoring effect to prevent delamination in the absence of the carbon fiber fillers. However, in the 5 wt. All these methods lead to the development of a rough surface that increases the mechanical anchorage between the substrate and the coating applied, making the primer more efficient in any 5% milled carbon fiber grade. The square dimple laser-textured samples show localized deformation along the wear path, including some areas of primer pull-out and matrix exposure. Dimples encourage better mechanical interlocking, which provides an anchorage style to the coating with the surface. However, the deformation is more significant in the 0 wt.% milled carbon sample, which indicates that the optimal wear resistance is achieved with the combined effect of dimple texturing and carbon fillers. The circular dimple laser-textured surfaces demonstrate a clear wear morphology, including detachment of the primer coat, matrix, and localized fiber damage. This illustrates that the round dimple pattern promotes stress concentration at the interfaces, resulting in coating delamination and fiber–matrix degradation during sliding contact. In the reference sample, with no laser texturing, the primer is fully pulled out, and the coating is completely removed from the matrix surface. This also confirms that the laser surface modification treatment exhibits an obvious improvement in the coating adhesion and wear resistance. The wear track analysis suggests that laser texturing increases the adhesion between the primer coat and the composite matrix, resulting in less matrix peeling. The effectiveness of these various laser patterns differs, with the square dimple texture showing the best mechanical interlocking and wear resistance properties, especially when combined with milled carbon fiber fillers. It shows how surface texturing helps improve the coating adherence to the substrate and, thus, reduces its wear rate values.

**Figure 11:**
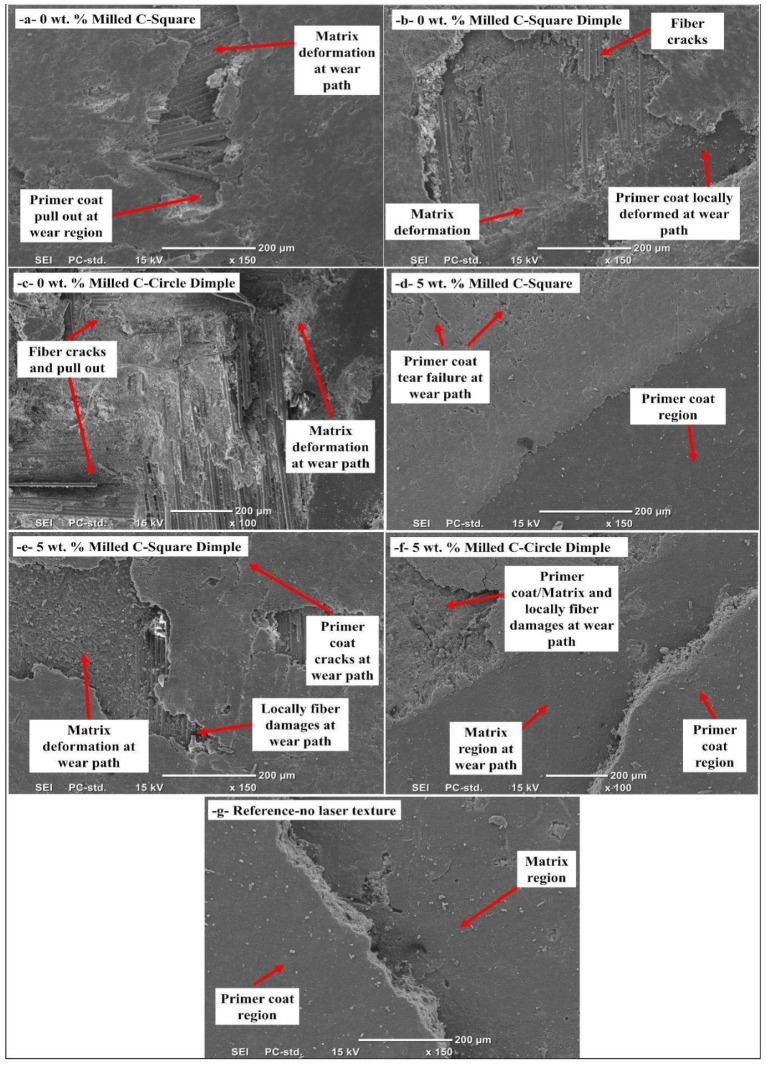
Wear surface of primer-coated and various laser-textured composite samples with 0% and 5% milled carbon fiber-filled (150×).

In Figure 11, detailed images (150×) taken from the wear track area formed after the pin-on-disc wear tests of composite samples on which different laser texturing was created on the surface (5 repeated shots) and then primer-coated, are shown. On the surface of the composite sample to which laser treatment was not applied, the primer coating and matrix area were clearly exposed. Especially, the presence of primer coating was not observed in the wear path area formed by the indenter tip. In the samples with laser texturing, it was observed that the square pattern showed positive adhesion, and despite the formation of local deformation in the primer coating in the wear path area, the deformation did not reach the surface of the composite material. In addition, in the samples with square dimples and circle dimple textures, it was determined that the damage occurred locally in the form of matrix and fiber breakage, but this deformation did not spread to the entire wear path area. It was determined that the primer coating adhered well to the material surface, especially with the laser texturing process, and that it removed some fibers together with the matrix from the surface of the composite material with the indenter effect. Considering that the main factor here is the compatibility of the primer coating with the composite material surface, it was determined that the primer coating was easily separated from the surface in samples that were not laser processed, and the coating did not hold on in the wear path area.

### 3.4. ANOVA Analysis

This study utilized a factorial experimental design method for a systematic analysis and evaluation of experimental data. Factorial design is a powerful method that can enable a concurrent examination of the impact of a range of factors on experimental outcomes. This method has a strong point, most importantly, for the evaluation of the impact of a range of factors at a range of levels and in a detailed analysis of outcomes. One of the strong points of a factorial design is its capability to expose complex relations produced through factors’ impact, in addition to the impact of individual factors. Besides, its efficiency in processing non-numerical experimental information enables a systemic analysis of both numerical and classification-data information. This method holds a strong point in terms of cost and time in generating a larger information yield with fewer experiments. In this study, ANOVA analysis was carried out to determine the individual and interactive contributions of the parameters used in the laser processing process on the output, rather than focusing on their classical assumptions. The effects of material type, laser texture structure, and number of laser pulses were analysed in detail by means of factorial experimental design, and it was observed that the model explained 100% of the total variance. The results of the analysis show that material type and texture interaction are the most determining factors, while the number of laser pulses has a relatively low effect. In this context, the study aimed to provide meaningful implications for the optimisation of the process. This study considered three factors in a 3-way factorial structure. In the factorial analysis, material and texture parameters were selected as categorical variables, while laser repetition was considered numerically. First, one of them is defined in terms of material type with two levels: no-carbon-added material (M0) and 5 wt.% carbon-added material in terms of weight (M5). Second, one is laser texture, and it is represented by T1, T2, and T3 notations. Third, one involves laser shot count with two levels: 5 and 10 laser shots. Interaction between these three factors and respective factors generated a 3-way factorial experimental scheme with 18 levels, and one can refer to them in Table 2. With such a scheme, individual and interactive effects of factors such as laser shot count, laser texture, and material type could be analyzed effectively. With a systematic approach in a study, experimental data could be analyzed effectively, and a reliable analysis could be performed for output. It is worth mentioning that inasmuch as assumptions of classical ANOVA, including residual normality and homoscedasticity, are not systematically tested in the current study because of the limited sample available as well as the categorical nature of a few variables, the analysis still strives to make provisional inferences about the interactive effects of the salient factors. However, subsequent studies are urged to incorporate assumption checking methods such as residual diagnostics or Levene’s test to further establish statistical rigor.

The ANOVA analysis shown in Table 3 examined the influence of three variables (material type, laser texture, laser pulse count) on the experimental results and the interactions between the variables. In this study, since two- and three-factor interactions were analysed in detail in addition to the main parameters, the error term, as seen in classical ANOVA analyses, did not appear as an independent component. Instead, the error value is distributed in the 2-Way Interactions and 3-Way Interactions sections, and the model explains the entire variance in a way that covers all variables and the relationships between them. The sequential sum of squares (SS) for the model was 232.917; it included the contribution of all components and their interactions in the experimental results. The model accounts for 100% of the total variance, meaning that all effects of factors and their interactions on the outcomes of the experiment are included. Although the model accounts for the full experimental variance since the factorial design accounts for all combinations of variables, we realize that perhaps the method might represent a potential drawback of overfitting, most critically in the case of the absence of an independent validation dataset. In upcoming research, the problem shall be countered by using data splitting or cross-validation methods. Analysis of the overall influence indicated that the contribution of linear effects to the total variance was 49.37%. The analysis showed that material type had the highest contribution (38.96%) of all factors. This discovery indicates that the type of material present has a material differential influence on the experiment connected to it. In terms of contribution, the texture variable contributed 8.66%, and laser repetition only 1.75%, which implies that laser repetition is a less significant feature than other variables. This accounted for 47.19% of the total variance in two-way interactions. The material type vs. texture interaction explained the most variance in the data at 39.28%. 4.33% of the variability was attributable to the interaction between material type and laser repetition, and 3.51% was due to the interaction between texture and laser repetition. Results such as these underline the significance of pairings of some factors in affecting experimental outcomes. This three-way interaction accounted for 3.51% of the total variance. However, the combination of material type, texture, and laser repetition affected the results of the experiment. This is especially important in experimental designs with multiple factors. The insufficiency of any error term implies a complete explanation of experimental data and a high-quality experimental design. In fact, the absence of an error component suggests that measurement errors might go unaccounted for, something that must be kept in mind while interpreting the results. ANOVA details individual and interactive effects of material, texture, and laser repetition on the experimental outcome. Of these factors, material type contributed the most, followed by the interaction of material type and texture. This confirms the strength of the experimental design and confidence in the collected data.

**Figure 12:**
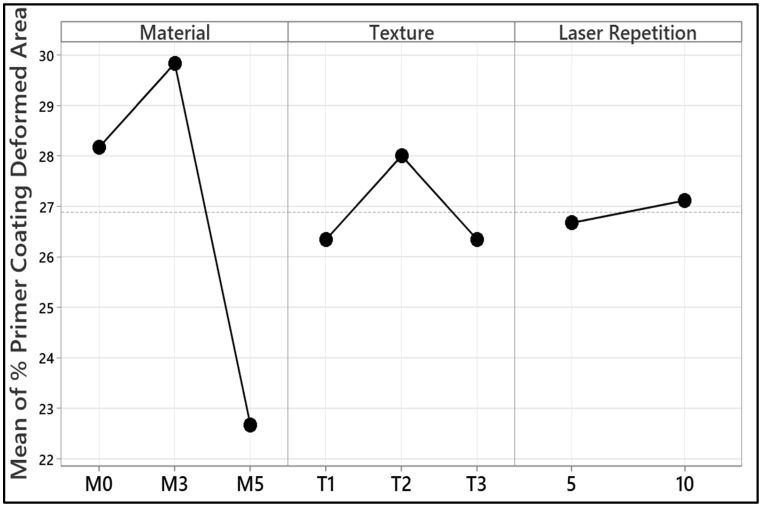
The main effects of material type (M0: carbon-free, M5: 5 wt.% carbon), laser texture (T1, T2, T3), and laser repetition (5 and 10 pulses) on the percentage of primer coating deformed area.

Figure 12 illustrates the contribution of material type, texture, and laser repetition to the percentage of primer coating deformed area. The most dominant influence arises from the material factor. The deformation percentage for the unmodified material (M0) is approximately 28%, whereas for the 5 wt.% adding material (M5), the deformation percentage significantly decreases to around 23%. This indicates that by increasing the reinforcement level, the deformation resistance of the primer coating will be higher. Looking into the texture factor, there is a significant difference between T1, T2, and T3. The deformation percentage for T2 is found to be highest, with slightly more than 27.5% in comparison to the other two textures (T1, T3), with around 25% deformation percentage. This variation confirmed that the laser texturing process affected the surface properties, which in turn had an impact on the material’s resistance to deformation. The laser pulse repetition makes a fairly minor difference by comparison. Each laser shot gives around 5% deformation time, so after five laser shots, it will get around 25% deformation, and for ten laser shots, it will get around 26% deformation. This implies that while more laser repetitions enhance deformation performance, it is not as strong a factor with respect to performance enhancement as is either the material type or the texture. As indicated in Figure 10, the most weighted factor in determining achievable deformation resistance is the material type, followed by the texture and laser repetition, which all play a role but to a lesser extent. The results shown above provide useful insights as to how each one of the factors influences the deformation performance of the primer coating.

## 4. Conclusions

This research showed that laser surface texturing greatly improves the wear resistance and adhesion of basalt fiber composites filled with milled carbon fibers. Square dimple textures along with a 5 wt.% carbon filler showed minimum deformation of the coating and optimal wear performance based on better mechanical interlocking. SEM and profilometry studies revealed laser-textured surfaces generating more average and shallow wear tracks, whereas the coefficients of friction identified the textured, carbon-filled surfaces with improved stability. The results from ANOVA showed surfacing texture and composition as the most dominant factors. The research emphasizes the applicability of laser texturing, especially with optimized reinforcement phases and patterns, as a promising tool for enhancing composite surface durability in aerospace and automotive sectors.

## Figures and Tables

**Table 1:** Factorial design of experiment parameter.

Input Parameter	Range
Material	0 wt.% milled carbon, 5 wt.% milled carbon
Texture	T1 (square) T2 (square dimple) T3 (circle dimple)
Repetition of textures	5, 10

**Table 2:** Eighteen-level experimental design, created with the factorial design method and measured % primer coating deformed area.

Material	Texture	Laser Repetition	Primer Coating Deformed Area (%)
M0	T1	5	24
M0	T1	10	30
M0	T2	5	33
M0	T2	10	34
M0	T3	5	23
M0	T3	10	25
M5	T1	5	22
M5	T1	10	22
M5	T2	5	20
M5	T2	10	22
M5	T3	5	27
M5	T3	10	23

**Table 3:** ANOVA analysis results for % primer coating deformed area.

Source	DF	Seq SS	Contribution	Adj SS	Adj MS
Model	11	232.917	100.00%	232.917	21.174
Linear	4	115	49.37%	115	28.75
Material	1	90.75	38.96%	90.75	90.75
Texture	2	20.167	8.66%	20.167	10.083
Laser Repetition	1	4.083	1.75%	4.083	4.083
2-Way Interactions	5	109.75	47.12%	109.75	21.95
Material × Texture	2	91.5	39.28%	91.5	45.75
Material × Laser Repetition	1	10.083	4.33%	10.083	10.083
Texture × Laser Repetition	2	8.167	3.51%	8.167	4.083
3-Way Interactions	2	8.167	3.51%	8.167	4.083
Material × Texture × Laser Repetition	2	8.167	3.51%	8.167	4.083
Total	11	232.917	100.00%		

## Data Availability

The datasets presented in this article are not readily available because the data are part of an ongoing study. Requests to access the datasets should be directed to Corresponding Author.

## Abbreviations

The following abbreviations are used in this manuscript:

SEM	Scanning electron microscope
FRPCs	Fiber-reinforced polymer composites
NFRCs	Natural fiber-reinforced composites
CFRPs	Carbon fiber-reinforced polymer composites
ANOVA	Analysis of Variance
